# Medical Students Abroad

**Published:** 1859-03

**Authors:** 


MEDICAL STUDENTS ABROAD.—The number of students who took out inscriptions for 1858–59, in Paris, is 1065, an increase of thirty-eight over last year. Louis Napoleon has determined to elevate the standard of requirements of those entering the medical profession of France, and has, therefore, issued an imperial decree that after April, 1859, all students must be Bachelors of Arts. There are 478 students attending the University of Edinburgh, showing an increase over last year's session.

